# A commentary on ‘Complex causal association between genetically predicted 731 immunocyte phenotype and osteonecrosis: a bidirectional two-sample Mendelian randomization analysis’

**DOI:** 10.1097/JS9.0000000000001442

**Published:** 2024-04-11

**Authors:** Xingcheng Zhu, Benlin Huang, Junxian Zhao, Chunqiong Yin, Xiangyun Li, Jieming Zuo, Junhao Chen, Hongping Bao

**Affiliations:** a Department of Clinical Laboratory, The Second People's Hospital of Qujing City Qujing; b Department of Gastrointestinal Surgery, The Second People’s Hospital of Qujing City, Qujing; c Department of Urology, The Second Affiliated Hospital of Kunming Medical University; d Department of Urology, 920th Hospital of Joint Logistics Support Force of Chinese People’s Liberation Army, Kunming, Yunnan Province, People’s Republic of China

Highlights

- Conclusions based solely on a single disease outcome database may lack credibility.
- To enhance the robustness of the conclusions, it may be considered to integrate osteonecrosis outcomes from multiple databases and perform a meta-analysis of single nucleotide polymorphism data; subgroup analysis could be beneficial for a deeper understanding given the multifactorial etiology of the disease.
- Further adoption of multivariable Mendelian randomization methods may aid in identifying exposure factors that can directly affect disease outcomes, apart from other immune cells.
- Sensitivity analyses should also incorporate MR-PRESSO, and after conducting two-sample Mendelian randomization analysis, it is necessary to implement false discovery rate (FDR) correction to reduce the risk of false positives.

*Dear Editor*,

We carefully read the article by Li *et al*.^1^ published in the International Journal of Surgery where they employed Mendelian randomization (MR) to assess the causality between 731 types of immune cells and osteonecrosis. The main results indicated a positive correlation between 8 immune cells and osteonecrosis, and a negative correlation with 18, with no reverse causality evident in reverse MR. These findings contribute to our understanding of the pathogenesis of osteonecrosis and how it might be prevented. We commend the authors for their outstanding work and innovative ideas. However, we would like to share some thoughts on this topic.

Although MR provides an effective alternative method to estimate the causal effect of exposure on outcomes using genetic variants as instrumental variables, especially when randomized controlled trials are not feasible, conclusions derived solely from a single disease outcome database may be limited and could lead to bias. Such limitations might interfere with accurate causal inference. The use of osteonecrosis data from genome-wide association studies (GWAS) databases suggests that incorporating osteonecrosis data from different databases, such as the UK Biobank, for independent validation could be a strategic improvement. Moreover, conducting a meta-analysis of single nucleotide polymorphism data across databases integrates data from multiple studies, increasing sample size and statistical power, yielding more stable and broadly applicable conclusions. Further, the IEU online database reveals the existence of multiple subtypes of osteonecrosis, such as drug-induced osteonecrosis (finn-b-OSTEON_DRUGS), highlighting the disease’s diverse etiology. Therefore, a subdivided study of drug-induced osteonecrosis to explore the role of the same immune cells may offer more targeted insights for clinical treatment.

In their study, the authors identified several immune cell types from 731 variants that have protective or promotive effects on the disease. While this preliminary study has revealed their correlation with disease outcomes, we believe these findings do not comprehensively explain how these immune cells independently affect specific health outcomes. With multiple exposures meeting the criteria for causality (*P*<0.05), we advocate for the use of more refined analysis techniques to further investigate the independent actions of these immune cells. The Multivariable Mendelian Randomization (MVMR) approach allows for the consideration of the effects of various protective and promotive immune cells, associating them with disease outcomes through MR analysis, while assessing the independent effect of single exposures on disease outcomes, controlling for other related exposures. This method is particularly suited to studying factors that directly impact health outcomes in the context of multiple exposures and interactions, providing deep insights for immune-cell-based treatments and preventive strategies, and laying the groundwork for further development of drug targets.

Lastly, MR-PRESSO is an essential method for MR analysis, offering an alternative perspective to detect pleiotropy in two-sample MR, allowing for the exclusion of specific single nucleotide polymorphisms and re-examination for pleiotropy. Despite traditional *P*-value correction methods, such as Bonferroni correction, the accumulation of false positives could lead to unreliable results. In contrast, false discovery rate (FDR) correction provides a more balanced approach, reducing the risk of false positives while retaining sensitivity for true findings, which is particularly important in the study of complex biological mechanisms and disease correlations.

In summary, we express our gratitude and appreciation for the contributions made by Li *et al*. in exploring the causal relationship between immune cells and osteonecrosis. Their work provides preliminary insights into the relationship and role of immune cells in osteonecrosis and lays a solid foundation for future in-depth research and the development of targeted treatments. Our suggestions and concerns are presented to help further refine related research methodologies, to advance a deeper understanding and development of future treatment strategies.

## Ethical approval

Not applicable.

## Consent

Not applicable.

## Sources of funding

Not applicable.

## Author contribution

X.Z., B.H., and J.Z.: design, literature search, and writing; C.Y., X.L., and J.Z.: literature search and data collections; H.B. and J.C.: providing language polish.

## Conflicts of interest disclosure

The authors declare no conflicts of interest.

## Research registration unique identifying number (UIN)

Not applicable.

## Guarantor

Junhao Chen.

## Data availability statement

The journal requires authors to include in any articles that report results derived from research data to include a Data Availability Statement. Please confirm if any datasets generated during and/or analyzed during the current study are publicly available, available upon reasonable request, or if data sharing is not applicable to this article.

## Provenance and peer review

Not applicable.
